# Assessment of disinfectant and antibiotic susceptibility patterns and multi-locus variable number tandem repeat analysis of *Staphylococcus epidermidis* isolated from blood cultures

**Published:** 2018-04

**Authors:** Parisa Asadollahi, Fereshteh Jabalameli, Reza Beigverdi, Mohammad Emaneini

**Affiliations:** Department of Microbiology, School of Medicine, Tehran University of Medical Sciences, Tehran, Iran

**Keywords:** *Staphylococcus epidermidis*, Disinfectants, VNTR, Blood cultures, Multidrug resistant

## Abstract

**Background and Objectives:**

Variable number tandem repeat (VNTR) patterns and resistance against three commonly used hospital disinfectants [0.5% (w/w) chlorhexidine digluconate (CHG) and 75% (w/w) alcohol (A), CHG-A; Quaternary ammonium compounds (QACs) and biguanides (B), QAC-B; and 70% (w/w) isopropanol (ISP) and 0.25% (w/w) QACs, ISP-QAC], as well as frequently used antibiotics, were evaluated among 115 *Staphylococcus epidermidis* blood isolates recovered from a children’s hospital in Tehran, Iran.

**Materials and Methods:**

Multi-locus variable number tandem repeat analysis (MLVA) was performed using primers targeting 5 VNTR loci on the genome of *S. epidermidis* isolates. Micro-broth dilution method and detection of *qacA/B* and *smr* genes were carried out for evaluating resistance against the disinfectants.

**Results:**

Out of the 115 isolates, 115 (100%) and 113 (98.3%) were susceptible to linezolid and quinupristin/dalfopristin, respectively. A total of 55.7% of the isolates were found to be multidrug resistant (MDR). All isolates had MICs of CHG-A and ISP-QAC of 8 folds lower and MIC of QAC-B 6 folds lower than that suggested by the manufacturers. The genes *qacA/B* and *smr* were found in 28 (24.3%) and 14 (12.2%) isolates, respectively. MLVA typing of the *S. epidermidis* isolates resulted in 106 VNTR patterns and 102 MLVA types for the 112 *S. epidermidis* isolates, considering that 3 were not typeable.

**Conclusion:**

MLVA typing of *S. epidermidis* isolates show a great diversity and that the isolates are still susceptible to the concentrations of disinfectants recommended for use by the manufacturers. In addition, the relatively high percentage of the MDR *S. epidermidis* isolates could cause MDR infections and act as reservoirs to transfer resistance determinants to *S. aureus* population. Therefore, it is important that suitable infection control strategies are employed to avoid the distribution of MDR isolates between personnel and patients in this medical centre.

## INTRODUCTION

*Staphylococcus epidermidis* is an opportunistic pathogen which is responsible for a large diversity of nosocomial infections, especially in patients with implanted medical devices and/or impaired immunity (1, 2). Cross-infections due to multi-resistant *S. epidermidis* are frequent in specific wards including neonatal intensive care units (NICU) (1) and contribute to 31% of all neonatal infections and 73% of neonatal bacteremia worldwide. Therefore, neonates often represent a particularly high-risk group for infections caused by this bacterium (3). Multilocus variable number tandem repeat analysis (MLVA) is a method that targets variable number tandem repeat (VNTR) loci on the genome and relies on the detection of different copy numbers of repeated sequences arrayed in tandem. MLVA has been proven to have a discriminatory capacity similar to that of pulsed-field gel electrophoresis (PFGE) and to be a promising, rapid and cost saving typing method for the epidemiological analysis of *S. epidermidis* (1, 4). In recent years, a considerable increase in the use of disinfectants and antiseptics in hospital settings has imposed a selective pressure for the emergence of disinfectant resistant microorganisms (5, 6). Quaternary ammonium compounds (QACs) and cationic biocides such as chlorhexidine are of the most frequently used disinfectants for decontamination of surfaces, disinfection of personnels’ hands and the treatment of patients colonized by pathogenic bacteria (6). Although the exact mechanism of resistance to disinfectants still remains uncertain, the *qacA, qacB* and *smr* genes have been considered as important determinants of resistance to QACs and other biocides by many authors (5–7). Despite the growing importance of *S. epidermidis* as a cause of hospital-acquired infections, there are still limited information regarding the epidemiology of this bacterium in hospital settings in Iran and other parts of the world. Therefore, the purpose of this study was to investigate the VNTR patterns as well as resistance against the three commonly used disinfectants among *S. epidermidis* blood isolates recovered from a children’s hospital in Tehran, Iran.

## MATERIALS AND METHODS

### Bacterial strains.

A total of 115 *S. epidermidis* isolates were included in this study. The isolates were recovered from blood cultures of children during April 2013 to August 2014 and epidemiological information was retrieved from admission notes of each clinical specimen. The samples were non-repetitive meaning that each patient was sampled only once. However, among the 115 isolates, 14 were obtained from 6 patients, so that 3 isolates with different colonies were recovered from one patient and 2 isolates of different colonies from each of the other 5 patients. Later on, antibiogram tests and other discriminating variables were analyzed to evaluate the uniqueness of these isolates. All the isolates were identified as *S. epidermidis* species using conventional biochemical tests (8) and confirmed by the detection of *nuc* gene which is an intrinsic and species specific gene among *S. epidermidis* isolates (9).

### Susceptibility testing.

Antibiotic susceptibility was performed by disk agar diffusion method for erythromycin, clindamycin, synercid (quinupristin/dalfopristin), linezolid, ciprofloxacin, rifampin, gatifloxacin, tetracycline, co-trimoxazole, and gentamicin according to the Clinical and Laboratory Standards Institute (CLSI) recommendations (10). Isolates exhibiting a resistance phenotype to at least four different classes of antibiotics were considered as multidrug resistant (MDR) (11). The minimum inhibitory concentrations (MICs) of vancomycin and oxacillin were determined by micro-broth dilution method according to the CLSI recommendations (10).

### MICs of disinfectants.

Commonly used disinfectants in a children’s hospital in Tehran, were obtained in commercial preparations. These disinfectants were: 0.5% (w/w) chlorhexidine digluconate (CHG) and 75% (w/w) alcohol (A) [CHG-A] a product used for the decontamination of personnel hands; Quaternary ammonium compounds (QACs) and biguanides (B) [QAC-B] for the disinfection of surfaces and floors; and 70% (w/w) isopropanol (ISP) and 0.25% (w/w) QACs [ISP-QAC] which disinfects medical devices.

MICs of the three disinfectants were determined by serial 2-fold broth micro-dilution of the disinfectants in Mueller-Hinton broth (Conda, Spain) which was, following autoclaving, added with the reducing dye triphenyltetrazolium chloride (TTC) (Sigma Aldrich, USA) according to the recommendations of the company. TTC was used for detection of microbial growth as a means of TTC reduction and a color change from colorless to red. Each microdilution was inoculated with 50 μl of 1.5 × 10^6^ CFU/mL of the overnight cultures in Brain Heart Infusion (BHI)-blood base agar. The microplates were then incubated at 37°C for 24 h and the concentration of the disinfectant that totally inhibited the growth of the microorganisms was recorded as the MIC.

### Detection of *qac* genes.

Colonies from an overnight culture on BHI-Blood base agar were suspended in 150 μl sterile water, vortexed for 1 minute, put in 100°C water bath for 15 minutes and then centrifuged 10 minutes at 12000 rpm, as previously described (12). Supernatant, containing DNA, was then used in PCR assay. PCR was carried out for detection of *qacA/B* and *smr (qacC/G)* genes using the primers previously published (13) (Table 1). The PCR mixture contained 5 μl of PCR master mix (Amplicon, Denmark), 0.25 μl of each forward and reverse primer, 4 μl of sterile water and 2.5 μl of the template DNA to make a final volume of 12.5μl. The PCR condition was as follows: DNA denaturation at 94°C for 5 minutes, followed by 30 cycles of 94°C for 30 s, 55°C for 30 s (50°C for 30 s for *smr* gene) and 72°C for 30 s. A final extension step at 72°C for 5 min was applied at the end.

**Table 1:** The primer sequences for MLVA typing and detection of disinfectant resistance genes in *S. epidermidis* isolates

**Genomic locus**	**Primer sequence 5′-3′ (forward/reverse)**	**Reference**
SE2101	TTCATTGTCCCCTGTCTTCT/TCGATCCTGGTAAAGCGATTA	
SE0331	GCTGATGGGGAAGAAGTTCA/AACGCTCCTAAACCTGCAAA	
I	AGGCCCAAATAAAAAGCAAA/AACTGACGCTCCAGGAGAAG	4
SE1632	TTTCCGGTATGTGAACCCTTA/TGACACTAGTCGCACAGGAA	
SE2395	GGCCATATAGACCTGGCTTG/AGATGCTGATGGGGAAGATG	
*qac A/B*	GCTGCATTTATGACAATGTTTG/AATCCCACCTACTAAAGCAG	15
*smr (qac C/D)*	ATAAGTACTGAAGTTATTGGAAGT/TTCCGAAAATGTTTAACGAAACTA	

### MLVA typing.

PCR, involving the five previously investigated VNTR primers was performed as described by Johansson et al. (4) (Table 1). PCR mixtures contained 10μl of PCR master mix (Amplicon, Denmark), 0.5μl of each forward and reverse primer, 9 μl of sterile water and 5 μl of the template DNA to make a final volume of 25 μl. Amplification of DNA fragments occurred with an initial denaturation at 95°C for 10 min and then cycling at 95°C for 1 min, 55°C for 1 min, and 72°C for 1 min for 30 cycles, with a final extension step at 72°C for 5 min. PCR amplification products were mixed with the KBC power load dye (GelRed Nucleic Acid Gel Stain, 10,000X in water, Kawsar Biotech Co., Tehran, Iran) and were analyzed for size variation on 1% agarose gel. The sizes of the PCR amplification products obtained were translated to copy numbers for each isolate using the formula: Copy number (bp) = [Amplicon size (bp) – Offset size (bp])/Repeat size (bp) (14), where amplicon size is the size of the PCR products on the gel, offset size is the size of the primers, and repeat size is the size of the tandem repeats in each VNTR locus. VNTR analysis was carried out according to two bands difference to designate a new MLVA type, considering that a selected isolate was used as the reference pattern. Each distinct type was assigned an arbitrary number, the most common type being allocated as type 1.

### Statistical analysis.

Data were analyzed using SPSS version 16 software. Isolates were grouped according to the presence/absence of the *qacA/B* and *smr* genes as well as MDR/susceptible phenotype and unpaired two-tailed t-test and Fisher exact tests were used to investigate the correlation between MICs of disinfectants and resistance genes as well as resistance phenotype. *P* values≤0.05 were considered significant.

## RESULTS

### Clinical data.

The distribution of the 115 *S. epidermidis* isolates among different wards of a children’s hospital in Tehran is demonstrated in Table 2. Eighty four percent of the isolates were collected from the emergency, infections disease, NICU and oncology wards as well as from outpatients. Out of a total of 64 MDR isolates, 56 (87.5%) belonged to the aforementioned wards. No association seemed to be present between the frequency of MDR isolates and a specific ward in the medical centre.

**Table 2:** Distribution of *S. epidermidis* isolates among different wards

	**Number (%)**		

**Ward**	**Isolates**	**MDR^3^**	**S^4^**
Emergency	27 (23.5)	16 (59.3)	11 (40.7)
OP^2^	22 (19.1)	14 (63.6)	8 (55.3)
Infectious disease	21 (18.3)	9 (42.9)	12 (37.3)
NICU^1^	20 (17.4)	14 (70)	6 (30)
Oncology	7 (6.1)	3 (42.9)	4 (57.1)
Gastro-enterology	5 (4.3)	1 (20)	4 (80)
Nephrology	4 (3.5)	2 (50)	2 (50)
Heart	2 (1.7)	1 (50)	1 (50)
Rheumatology	3 (2.6)	2 (66.7)	1 (33.3)
Neurology	2 (1.7)	2 (100)	0 (0)
Surgery	2 (1.7)	0 (0)	2 (100)
Total	115 (100)	67 (58)	48 (41.7)

1 Neonatal intensive care unit,

2 Out patient,

3 Multidrug resistant,

4 Susceptible

Among the 115 isolates, 13 were obtained from 6 patients, so that 3 isolates were recovered from one patient and 2 from each of the other 5 patients. Properties of these 13 isolates are shown in Table 2. Isolates showed different combinations of antibiotic and disinfectant resistance profiles, *qacA/B* and presence of *smr* gene and MLVA types (Table 3).

**Table 3:** Properties of the 14 isolates obtained from the blood samples of 6 patients.

**Patient**	**Isolate ID**	**Resistant against**	**Intermediate to**	**Antibiotic resistance phenotype**	***qacA/B***	***smr***	**MIC of disinfectants % (w/w)**			**MLVA type**

							**CHG-A**	**ISP-QAC**	**QAC-B**	
1	1a	RIF, OXA	TE	S	-	-	0.0976	0.195	0.003	11
	1b	ERY, CLI, GM, OXA	TE, CIP	S	-	-	0.0244	0.195	≤0.000375	Non-typeable

2	2a	TS, CLI	-	S	+	-				17
	2b	TS, ERY	-	S	-	-	0.006	0.0244	≤0.000375	18
	2c	TS	VAN	S	-	-	0.006	0.0122	≤0.000375	19

3	3a	TE, TS, OXA	VAN	MDR	-	-	≤0.000375	≤0.000375	≤0.000375	27
	3b	ERY, TE, CTS, OXA	-	MDR	-	-	0.00075	≤0.000375	≤0.000375	28
							0.00075	≤0.000375	≤0.000375	

4	4a	CIP, GAT, TS, OXA	VAN	MDR	-	+	0.006	0.0976	≤0.000375	31
	4b	CIP, GAT, TS, OXA	-	MDR	-	+	0.0122	0.195	≤0.000375	32

5	5a	ERY, CLI, CIP, GAT, TS, GM, OXA	-	S	-	-	0.0122	0.0488	≤0.000375	36
	5b	CLI, CIP, GAT, TS, OXA	VAN	MDR	+	-	0.0976	0.0244	≤0.000375	37

6	6a	TS	-	S	-	+	0.0122	0.0244	≤0.000375	41
	6b	ER, CLI, CIP, RIF, GAT, TE, TS, GM, OXA	VAN	MDR	+	-	0.0244	0.0976	≤0.000375	42

ERY: erythromycin, CLI: clindamycin, SYN: synercid (quinupristin/dalfopristin), CIP: ciprofloxacin, RIF: rifampin, GAT: gatifloxacin, TE: tetracycline, TS: co-trimoxazole, GM: gentamicin, OXA: oxacillin, VAN: vancomycin; MDR: multidrug resistant

CHG-A: 0.5% (w/w) chlorhexidine digluconateand 75% (w/w) alcohol; ISP-QAC: 70% (w/w) isopropanol and 0.25% (w/w) Quaternary ammonium compounds (QACs); QAC-B: QACs and biguanides

### Antibiotic susceptibility.

Antibiotic susceptibility profiles showed that, out of the 115 isolates, 115 (100%) and 113 (98.3%) were susceptible to linezolid and synercid, respectively. Rifampin was the next most effective antibiotics to which 108 (93.9%) isolates were sensitive (Table 4). On the other hand, erythromycin and co-trimoxazole were the least effective antibiotics against the *S. epidermidis*, so that 81 (70.4%) and 94 (81.7%) isolates were resistant to these antibiotics respectively. The MIC_50_ and MIC_90_ of vancomycin and oxacillin were 2 and 8 μg/ml and 4 and 64μg/ml, respectively; having MIC ranges of 1–64 μg/ml for vancomycin and 0.12–128 μg/ml for oxacillin.

**Table 4:** Frequency of antibiotic resistance among 115 *S. epidermidis* isolates

**Antibiotic**	**Number (%)**		

	**Susceptible**	**Intermediate**	**Resistant**
Linezolid	115 (100)	-	0 (0)
Synercid	113 (98.2)	-	2 (1.8)
Rifampin	96 (83.5)	-	19 (16.5)
Gatifloxacin	92 (80)	-	23 (20)
Gentamicin	79 (68.7)	2 (1.7)	34 (29.6)
Ciprofloxacin	74 (64.4)	1 (0.87)	40 (34.8)
Tetracycline	72 (62.6)	2 (1.7)	41 (35.7)
Clindamycin	48 (41.7)	2 (1.7)	65 (56.5)
Erythromycin	34 (29.6)	-	81 (70.4)
Co-trimoxazole	20 (17.4)	1 (0.87)	94 (81.7)

### MIC of disinfectants.

The MIC_50_ and MIC_90_ for CHG-A, ISP-QAC and QAC-B are shown in Table 5. CHG-A is recommended by the manufacturer to be used without dilution, giving a final concentration of 0.5% (w/w) chlorhexidine and 75% (w/w) alcohol. In this study, all the isolates had MICs of ≤0.39% (w/w) of CHG-A. ISP-QAC is also recommended for use without dilution and all the isolates showed MICs lower than or equal to 0.39% (w/w) ISP-QAC.

**Table 5:** MIC_50_ and MIC_90_ of CHG-A, ISP-QAC and QAC-B

	**Disinfectants MIC % (w/w)**		**MIC range% (w/w)**

	**MIC_50_**	**MIC_90_**	
CHG-A	0.012	0.195	0.000375-50
ISP-QAC	0.024	0.098	0.000375-50
QAC-B	≤0.00038	0.0015	0.000375-50

CHG-A: 0.5% (w/w) chlorhexidine digluconate and 75% (w/w) alcohol; ISP-QAC: 70% (w/w) isopropanol and 0.25% (w/w) Quaternary ammonium compounds (QACs); QAC-B: QACs and biguanides

The manufacturer suggests that at least 0.5% (w/v) solution of QAC-B should be used to disinfect surfaces and floors and all the isolates showed MICs of ≤0.006% (w/w) QAC-B. Among the 3 agents, QAC-B was the most effective disinfectant having a MIC_50_ and MIC_90_ of 0.00038% (w/w) and 0.0015% (w/w), respectively.

### *qac* and *smr* resistance genes.

Out of the 115 *S. epidermidis* isolates, *qacA/B* and *smr* genes were found in 28 (24.3%) and 14 (12.2%) isolates, respectively. The isolates carrying *qacA/B* gene had significantly higher MICs of CHG-A (*p* = 0.0316) and ISP-QAC (*p* = 0.0053) than those without *qacA/B* genes. There was no significant difference in the MIC of QAC-B between *qacA/B*-positive and -negative isolates (*p* = 0.4168).

No significant difference was found in the MICs of CHG-A, ISP-QACo and QAC-B between *smr*-positive and -negative isolates (*p* = 0.9875, 0.0572, and 0.0977, respectively).

### MLVA typing.

MLVA typing of the *S. epidermidis* isolates resulted in 106 VNTR patterns for the 115 *S. epidermidis* isolates, considering that 3 were not typeable.

MLVA typing produced 102 total types, in which 16 isolates were classified into 6 different types and the other 96 isolates were each classified as a separate MLVA type (Table 6). As demonstrated in this table, 5 out of the 16 isolates, exist in the most frequent MLVA type, 3 isolates in the second common type and 2 isolates in each of the 4 remaining MLVA types.

**Table 6:** The most common VNTR types of *S. epidermidis* isolates.

**Types**	**Repeat copy no. at tandem repeat locus**					**No. of isolates in each type**

	**SE2101**	**SE0331**	**I**	**SE1632**	**SE2395**	
1	6.00	17.00	8,6*	20.00	87.00	5
	6.00	17.00	6.00	23.00	87.00	
	6.00	20.00	6.00	×	87.00	
	6.00	20.00	6.00	23.00	81.00	
	6.00	53, 42, 20, 14*	6.00	23.00	×	

2	×	20.00	8,6*	×	×	3
	×	20.00	8,6*	×	×	
	×	20.00	8,6*	×	×	

3	87.00	48.00	8.00	26.00	81.00	2
	87.00	48.00	8.00	26.00	81.00	

4	×	39.00	8.00	×	94.00	2
	6.00	53, 48, 20, 14*	8,6*	×	×	

5	×	×	8,6*	24.00	×	2
	×	×	8,6*	24.00	×	

6	×	53.00	6.00	26.00	×	2
	×	53.00	6.00	26.00	×	

* Some specific tandem repeat loci occurred more than once along the genome in some isolates

## DISCUSSION

In this study, the effect of three disinfectants commonly used in a children’s hospital in Tehran was tested for the 115 *S. epidermidis* isolates. All isolates had MICs of CHG-A and ISP-QAC of 9 fold lower and MIC of QAC-B 8 fold lower than that suggested by the manufacturers, which suggest that in case the disinfectants are used according to the manufacturers’ instructions, the growth of 100% of the isolates would be inhibited.

MICs of CHG-A and ISP-QAC were significantly higher among *qacA/B* positive isolates compared with *qacA/B* negative isolates, whilst there was no significant difference in the MIC of QAC-B between these two groups of isolates. This can suggest that the presence of *qacA/B* gene might be an important determinant for the resistance of *S. epidermidis* isolates against CHG-A and ISP-QAC, whilst mechanisms other than *qacA/B* gene might contribute to resistance against QAC-B. No significant difference was also seen in the MICs of the three disinfectants among *smr* positive and negative isolates, which would also suggest the lack of necessity of *smr* gene in resistance against these disinfectants.

In this study, no significant difference was found in the MIC of CHG-A, ISP-QAC and QAC-B between the MDR and susceptible organisms. This result is in contrast with that reported in other studies which suggest that resistance to antibiotics and antiseptics may closely interface (6, 15–17). This may suggest that the genetic determinants of resistance to the antibiotics and disinfectants used in this study are not linked or the mechanisms of resistance to these agents are different (7). Based on the visual analysis of the MLVA patterns, 102 types were found among the 115 isolates which shows a great diversity of MLVA types among *S. epidermidis* isolates, as also described in a study by Francois et al. (1).

Among the 115 *S. epidermidis* isolates, 13 were obtained from 6 patients. These isolates showed different combinations of MLVA types, *qacA/B* and *smr* gene presence, and antibiotic and disinfectant resistance profiles, which suggests the presence of distinct isolates in those specified patients. This observation may be due to non-appropriate infection control strategies in a children’s hospital in Tehran, which may have caused the transmission of these isolates between personnel and patients. The significance of this remark is that since 55.7% of the isolates in this study are MDR, the entrance of adequate numbers of these isolates into the blood of a patient with low body resistance could cause MDR septicemic infections which are seriously life-threatening. In addition, a relatively high MDR level among *S. epidermidis* isolates in the current study, suggests their reasonable success in acquiring resistance determinants and the possibility to transfer these determinants into *Staphylococcus aureus* population (18), which could also be a major health concern. Therefore, it is important that suitable and standard hygiene policies are employed in a children’s hospital in Tehran to avoid the distribution of MDR isolates between personnel and patients. It is also worth mentioning that, considering the significance of MDR *S. epidermidis* in refractory infections, it is important that coagulase negative staphylococci are identified to the species level and that antibiotic resistance profiles are taken more seriously prior to antibiotic therapy against infections caused by these bacteria.

Taken together, the above observations indicate that MLVA typing of *S. epidermidis* isolates show a great diversity and that the isolates are still susceptible to the concentrations of disinfectants recommended by the manufacturers. In addition, the relatively high percentage of the MDR *S. epidermidis* isolates could cause MDR infections and act as reservoirs to transfer resistance determinants to *S. aureus* population. Therefore, it is important that suitable infection control strategies are employed to avoid the distribution of MDR isolates between personnel and patients in this medical centre.
